# Differences in the emotional conflict task between individuals with high and low social adjustment: An ERP study

**DOI:** 10.1371/journal.pone.0217962

**Published:** 2019-06-12

**Authors:** Yuan-Yan Hu, Jun-Cheng Zhu, Ying Ge, Wen-Bo Luo, Tian-Tian Liu, Xi Pu, on behalf of The Key Research Base for Humanities and Social Sciences of Chongqing

**Affiliations:** 1 Laboratory of Emotion and Mental Health, Chongqing University of Arts and Sciences, Chongqing, China; 2 Center for Mental Health Education, Southwest University, Chongqing, China; 3 School of Psychology, Jiangxi Normal University, Nanchang, China; 4 School of Education, Shanghai Normal University, Shanghai, China; Kochi University of Technology, JAPAN

## Abstract

To investigate the emotional conflict processing during the processing of emotional stimuli in individuals with different levels of social adjustment through developing an event-related potential (ERP) method, the study used positive words (happy), negative words (disgusted), positive faces and negative faces as experimental materials for a face-word Stroop emotional conflict task, which was completed by 34 participants. For the N2 component, there was a significant difference between the high and low social adjustment groups for the congruent condition; the low social adjustment group evoked more negative amplitude under the congruent condition. Under the incongruent condition, there was a marginally significant difference between the high and low social adjustment groups; the low social adjustment group evoked more negative amplitude under the incongruent condition. For the SP component, there were no significant differences for both the high and low social adjustment group between the congruent and incongruent conditions of emotional conflict. However, within the low social adjustment group, the incongruent evoked more positive amplitude. Our findings indicate that the difference in the emotional conflict process between individuals with high and low social adjustment mainly lies in the early processing stages of emotional information. That is, for both congruent and incongruent emotional stimuli, individuals with high social adjustment showed better emotional conflict monitoring, used less cognitive resources, and had a higher degree of automated processing than those with low social adjustment. During the later stages of emotional conflict processing, individuals with low social adjustment showed poorer conflict processing.

## Introduction

Social adjustment refers to the strong need and desire for individuals within a social environment to achieve social harmony [1, 2]. Social adjustment is not only a key condition in the measurement of an individual’s psychological maturity, it is also an important indicator for the measurement of their social maturity [3]. For instance, such social phenomena as irony in speech, satire, courtesy laugh are all examples of emotional conflicts with negative [4] and positive emotion stimuli [5]. Therefore, the ability to correctly identify the true meaning of irony etc. is crucial for successful social interactions and may be directly relevant to social adjustment. Furthermore, understanding the psychological and behavioral features of social adjustment in individuals will enable us to further elucidate its internal characteristics and patterns of development.

An individual’s level of social adjustment is intimately associated with their emotional perception and control [6]. Individuals with high social adjustment have good control over their own emotions and can rapidly maintain a balance with their surrounding environment. Conversely, individuals with low social adjustment are unable to quickly regulate their emotions, and have poor abilities in emotional perception and emotional control [7]. Otto, Doeringseipel, Grebe, and Lantermann [8] found that emotional perception and emotional control are key components of emotional intelligence (EI). EI is the ability to process emotional information, which encompasses the accurate evaluation of emotional information in one’s self and in others, the appropriate expression of emotions, and the ability to regulate emotions adaptively [9]. A large number of studies have found that EI is an important factor influencing an individual’s social adjustment. For example, EI has a significantly positive correlation with positive social adjustment, whereas it has a significantly negative correlation with negative social adjustment [10, 11]. That is, compared to individuals with low EI, those with high EI could more accurately recognize emotional information in them and in others, and thus were able to make positive adjustments based on their external and internal needs. This enabled them to maintain a favorable condition and harmonious relationships with the external environment, thereby allowing them cope more effectively in problematic situations and possess stronger adaptability [9, 12, 13].

The previous studies used the emotional Stroop paradigm to study different types of emotional disorders patients, and found that anxiety patients showed a processing advantage for threatening vocabulary information [14], while social phobia patients only show a slower response to fear-related vocabulary [15]. In contrast, the normal populations with different social adjustment have little research on the cognitive processing characteristics of emotional information.

Emotional conflict is a key research topic in the processing of emotional information, and has received increasing attention from researchers over the past decades [4, 6]. Emotional conflict refers to the interference of irrelevant emotional stimuli in current cognitive tasks [16, 17]. The face-word Stroop task is a classic experimental paradigm, which was often used to study emotional conflict. The paradigm involves overlapping emotional faces (positive and negative) and emotional words (positive and negative) to form stimuli, which are then grouped into the congruent and incongruent conditions. The face-word Stroop paradigm needs participants to ignore the color of the facial stimuli but judge the color of the word stimuli [18–21]. The face-word Stroop paradigm was conducted by several studies. For example, Egner, Etkin, Gale, and Hirsch adopted the face-word Stroop paradigm to study the neural systems and conflict resolution [22]. In addition, Strand, Oram, & Hammar investigate attention to and inhibition of emotional information [23]. Therefore, the Stroop paradigm was also adopted in our study to investigate the emotional conflict processing in different levels of social adjustment.

The face-word Stroop has been employed in many studies [19, 20, 24] to investigate emotional conflict, and the basic conclusions are that the reaction time for the incongruent condition is significantly higher than the congruent condition, while the accuracy of the incongruent condition is significantly lower than the congruent condition. This indicates that emotional faces will interfere with the participants’ cognitive processing task for emotional words, thus generating the emotional conflict Stroop effect [19, 20, 24].

The emotional Stroop effect is influenced by various factors, including cognitive styles [25], attention [26] and so on. In relevant studies, the cognitive processing of the emotional Stroop effect has been shown to activate certain brain mechanisms and to evoke multiple electroencephalography (EEG) components. For example, Etkin et al. [16], performed the face-word Stroop task and used fMRI to examine the neural mechanisms of emotional conflict. Their results showed that a high level of emotional conflict could be observed in the activity of the amygdala and prefrontal cortex, thus effectively dissociating emotional control from emotional resolution. Larson, Clayson, and Clawson [27] found that N2 belongs to the family of “conflict monitoring” components. In the classic Stroop paradigm, studies have found that the slow potential (SP) is a type of late slow wave component [27, 28] that is mainly associated with conflicts. Researchers believe that SP main reflects an individual’s emotional conflict processing in the emotional conflict task [29].

Therefore, it is not difficult to find that many studies have conducted questionnaire surveys to examine the relationship between emotional competence and social adjustment. In some previous studies, Tan [30] found that children’s emotionality and emotion control common effect its social adjustment. Another research found that emotion recognition and cognitive reappraisal can predict social maladjustment [31]. In other words, the previous studies told us that an accurate perception of others’ emotional states is related to a greater degree of social adjustment [10]. To our knowledge, however, no study has yet to investigate the cognitive features of individuals with different social adjustment levels when processing emotions. In order to clarify the relationship between social adjustment and emotion, the present study adopted event-related potentials (ERPs) which could provide precise information about the time dynamics of the brain to explore it. Comparing to traditional subjective self-report, Event-related potentials, which are closely related to people's emotions, have the advantages of millisecond high time resolution [32]. And it is very effective to accurately record the transient changes of different social adaptation groups in the process of emotional perception from the perspective of time, which is considered to be a very effective emotional research tools. An exploration of this issue will enrich the relevant theoretical research of social adjustment, while also serving as a reference for the modification of social adjustment in individuals.

Basing on previous studies, the scalp distribution of Stroop-related ERP components (N2 and SP) include two sites (i.e., frontal-medial and posterior parietal) [27]. According to the studies [27, 33], the frontal-medial negative component between 200 and 260 ms at sites FC1, FCZ, FC2, F1, FZ, F2, C1, CZ and C2, the posterior parietal positive component between 700 and 800 ms at sites Pz, P1, P2, POz, PO3 and PO4 were chosen for quantification analysis.

In summary, the present study aimed to employ the face-word Stroop paradigm and ERPs to investigate the cognitive features and the temporal characteristics of brain activity in individuals with different social adjustment levels when performing an emotional conflict task. We hypothesized that (1) N2 amplitude in response to congruent emotion expressions would be larger in low social adjustment group than in high one; (2) SP amplitude in response to emotion expressions would be not significantly difference between low social adjustment group and high one.

## Methods

The participants were informed of the study objectives before the experiment, and had given then written informed consent. This study had been approved by the Ethics Committee of the Institute of Education, Chongqing University of Arts and Sciences.

### Materials

#### China College Student Adjustment Scale (CCSAS)

The CCSAS compiled by Fang, Wo and Lin [34] was adopted to measure the individual’s social adjustment. This scale is composed of 7 dimensions, including interpersonal adjustment, learning adjustment, campus life adjustment, career adjustment, emotional adjustment, self-adjustment, and satisfaction, which constitute a total of 60 items. Each item was scored on a 1 (Disagree) to 5 (Agree) point scale, and a higher the total score represented a higher level of social adjustment. A previous study has shown that this scale has good reliability [34]. In this study, the Cronbach’s α of each dimension ranged between 0.65~0.82, and that of the overall questionnaire was 0.92.

#### Experimental materials for the emotional conflict task

**Fig 1:**
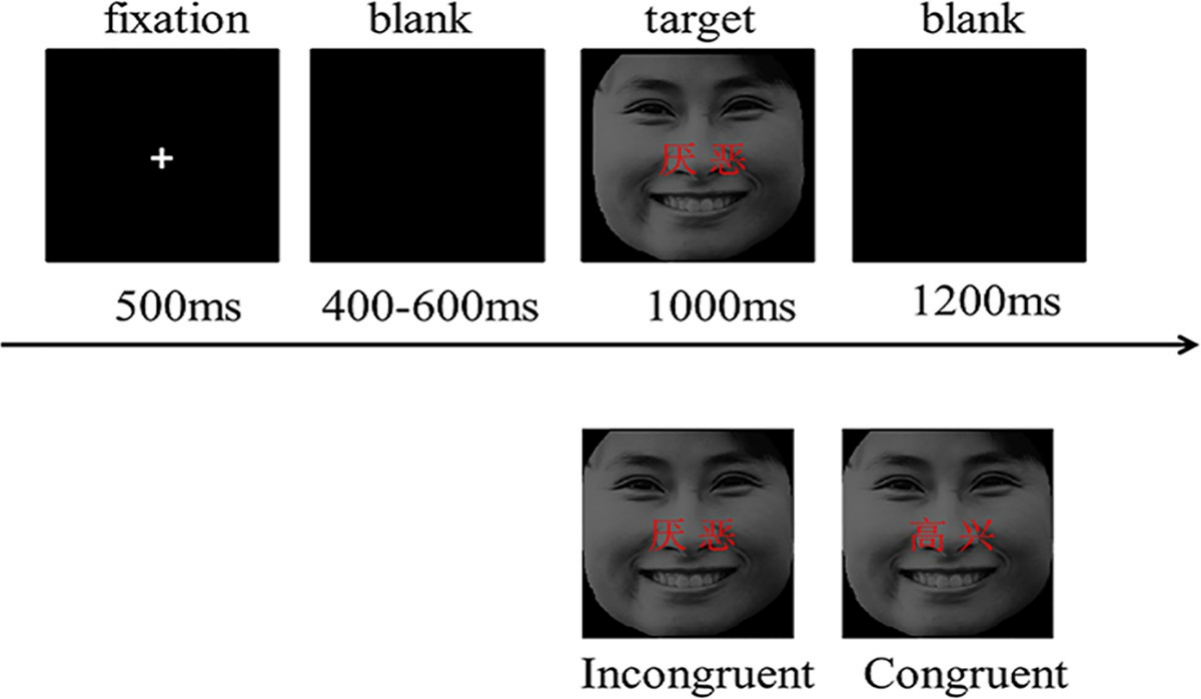
Example stimulus of each condition type and task paradigm.

Facial images were selected from the Chinese Affective Picture System (CAPS), consisting of 10 “Happy” facial images (five male and five female) and 10 “Disgusted” facial images (five male and five female) [35]. According to the literature[20], the two Chinese words “愉快” (means “happy”) and “厌恶”(means “disgusted”) were written in red on each facial images. The size of face is 9.17cm (horizontal) x10.28cm (vertical), and the word’s font size is 20 Song typefaces in red (Fig 1).

Finally, we got 40 facial images as experimental stimuli. Among them, the emotional conflict condition was when the facial expression was incongruent with the red Chinese characters; the reverse was the non-conflict condition. Another 5 “disgusted” and 5 “happy” facial photographs were selected and subjected to the same treatment to produce practice materials for the participants.

### Participants

The CCSAS was administered to 300 undergraduates in a university in Chongqing Undergraduates who scored the highest and lowest 27% were assigned to the high and low social adjustment groups. This was combined with their basic situation (whether they volunteered to participate in the experiment, whether they have participated in similar experiments before etc.) to select the final participants who met the requirements of the experiment. The high social adjustment group (total score ≥239 points) included 16 participants, and the low social adjustment group (total score ≤146 points) included 18 participants, giving a total of 34 undergraduates. The participants’ ages were between 19–24 years, with a mean age of 21.7 years and standard deviation of 0.93 years. Independent samples *t* test was performed on the total CCSAS scores of the two groups, and the results were that *t* (32) = 27.37, *p* < 0.001, *Cohen's d* = 9.68, which indicates that the participant screening was effective. The participants were all right-handed; had normal visual acuity or corrected visual acuity; did not have color vision deficiency or color blindness; had no physical or mental diseases; and had never participated in similar experiments. The participants were informed of the study objectives before the experiment, and had given then written informed consent. This study had been approved by Chongqing University of Arts and Sciences. The participants received a certain amount of remuneration after the experiment.

### Experimental design

A 2 (social adjustment: high vs. low) × 2 (emotional conflict: congruent vs. incongruent) two-factor mixed design was used for the experiment. Specifically, social adjustment was the between-group factor, and emotional conflict was the within-subject factor. The dependent variables were the accuracy and reactive time (RT) of the participants’ keypress, and the EEG data collected during the experiment.

### Experimental procedure

The experimental stimuli were presented using the E-prime 2.0 software. The stimuli were presented against a black background, and the participants’ RT and accuracy were recorded automatically by the computer. In the face-word Stroop task, a white fixation cross “+” was first presented in the center of the screen for 500ms, followed by a blank screen presented for a random duration between 300-500ms. Then, the stimulus was presented for 1000ms, and finally a blank screen was presented for 1200ms. The participant’s task was to rapidly and accurately judge the facial expressions of the stimulus (“disgusted” or “happy”), while ignoring the red emotional words (“disgusted” or “happy”) written on the nose of faces. If the facial expression was happy, then the participant pressed the “1” key with their right index finger. If the facial expression was disgust, then they pressed the “2” key with their right middle finger. The experiment consisted of 280 trials, divided into 7 blocks, with 40 trials in each block. The number of happy and disgusted expressions in each block was the same, and the order of presentation was randomized. There was a short break between each two block. The participants were given 32 practice trials before starting the formal experiment, in order to ensure the participants were quickly familiarized with the keypress and the experimental process.

### Data collection

The Brain Vision EEG recording and analysis software (Germany) was used record EEG data from a 64-electrode EEG cap according to the International 10–20 system. The horizontal electrooculography (HEOG) was recorded from electrodes positioned at the outer canthi of both eyes; the vertical EOG (VEOG) was recorded from electrodes above and below the right eye; the reference electrode was placed on the bilateral mastoid. All electrodes recorded had an impedance of below 5kΩ; the band-filter was 0.1-30Hz, and EEG signals with electrode voltage more than ±80uv were automatically removed.

### Statistics and analysis (behavioral and ERP data)

#### Behavioral data analysis

Statistical analysis was performed on the behavioral data using SPSS 11.5. Social adjustment (high vs. low) × emotional conflict (congruent vs. incongruent) two-factor repeated measures analysis of variance (ANOVA) was performed on the accuracy and RT data. 2.8% of data was lost after 3SD exclusion criteria.

#### ERP data analysis

The epoch for analysis was time-locked to between 200ms before stimulus presentation and 1000ms after stimulus presentation. The signal from the first 200ms before stimulus presentation was taken as the baseline. The EEG signals of trials with correct responses from both conditions were superimposed and averaged. The number of valid superpositions for the overall averaged ERP for each condition was no less than 90. With reference to past studies [33, 36], as well as the overall averaged and difference waveforms obtained in this study, we selected the FC1, FCZ, FC2, F1, FZ, F2, C1, CZ and C2 electrodes for statistical testing in the N2 (200-260ms) time window; and the Pz, P1, P2, POz, PO3 and PO4 electrodes for statistical testing in the SP (700-800ms) time window. Analysis was performed using social adjustment (high vs. low) × emotional conflict (congruent vs. incongruent) two-factor repeated measures ANOVA. For interaction effects, the LSD method was used to performed simple effects analysis, and Greenhouse-Geisser was used to correct the *P* values.

## Experimental results

### Behavioral results

The mean RT and accuracy of participants with high and low social adjustment are shown in S1 Table. Two-factor repeated measures ANOVA was performed on the RT and accuracy. In terms of RT, the main effect of emotional conflict was significant, *F*(1, 32) = 52.07, *p* < 0.001, *η2 p* = 0.62, whereby the RT of the congruent condition was significantly faster than the incongruent condition. The main effect of social adjustment was not significant, *F*(1, 32) = 0.53, *p* >0.05, *η2 p* = 0.08. The interaction effect of social adjustment and emotional conflict was not significant, *F*(1, 32) = 0.35, *p* >0.05, *η2 p* = 0.06. In terms of accuracy, the main effect of emotional conflict was significant, *F*(1, 32) = 47.67, *p* < 0.001, *η2 p* = 0.60, whereby the accuracy of the congruent condition was significantly higher than the incongruent condition. The main effect and interaction effect of social adjustment were both not significant.

### ERP results

**Fig 2:**
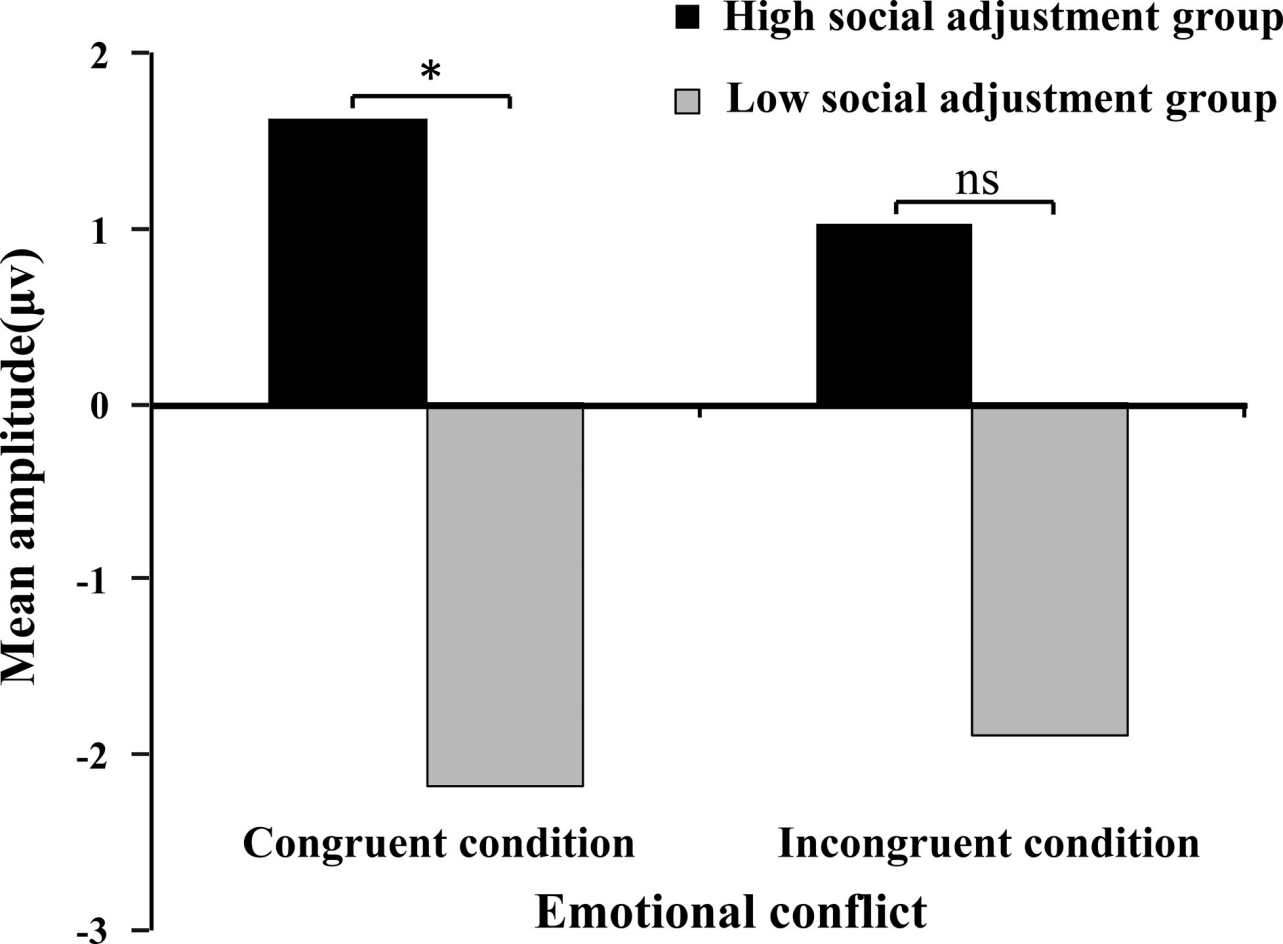
Interaction effect between social adjustment and emotional conflict for the N2 component.

For the N2 component, the main effect of emotional conflict was not significant, *F* (1, 32) = 0.47, *p*>0.05, *η2 p =* 0.02, while the main effect of social adjustment was significant, *F* (1, 32) = 4.80, *p*<0.05, *η2 p* = 0.13. The interaction effect of emotional conflict and social adjustment was marginally significant, *F* (1, 32) = 3.53, *p* = 0.07, *η2 p* = 0.10 (Fig 2).

**Fig 3:**
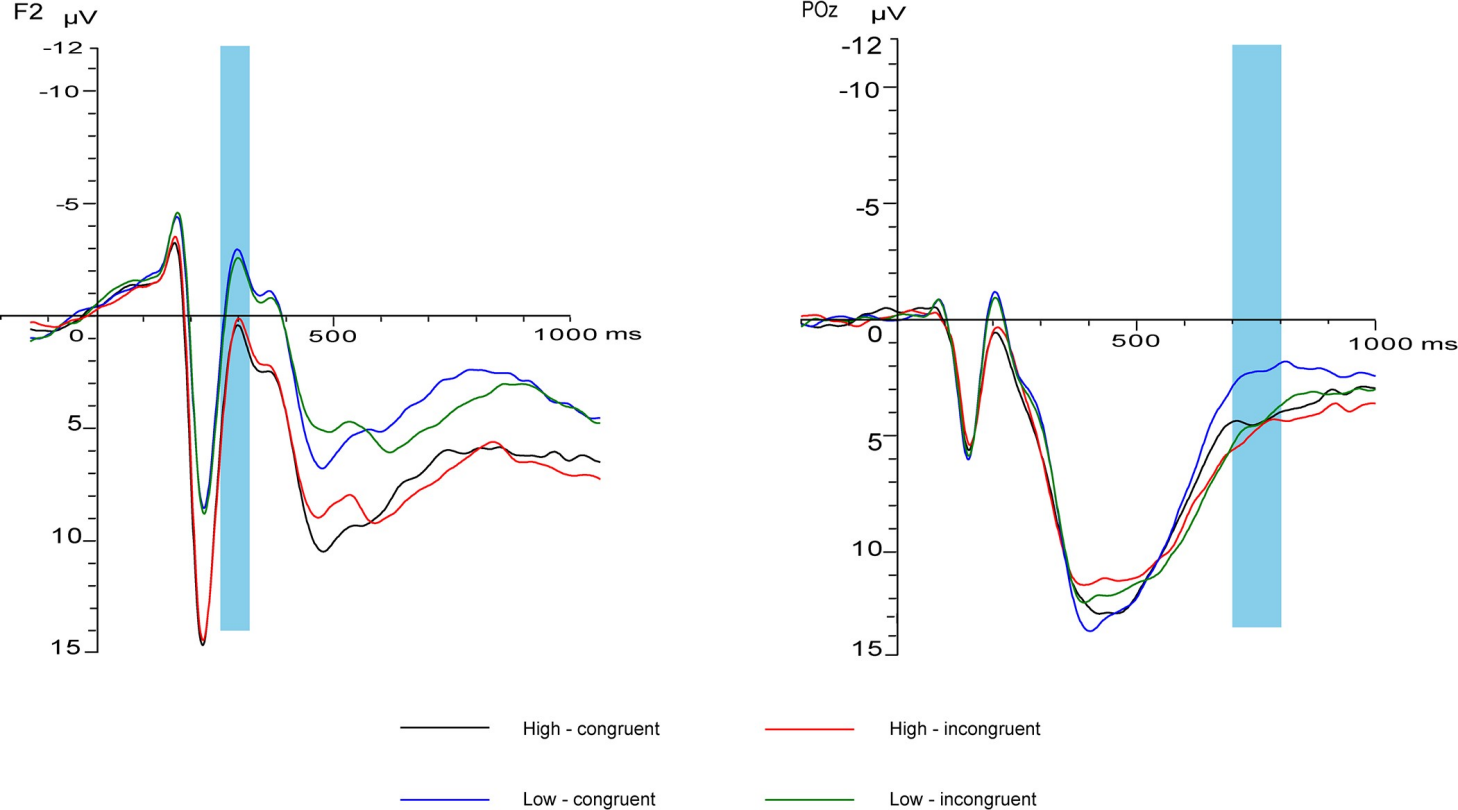
Emotional conflict effect of N2 & SP component shown in the fronto-central electrodes.

Simple effects analysis was performed on the interaction effect between emotional conflict and social adjustment. The results showed that under the congruent condition, there was a significant difference between the high and low social adjustment groups (*F* (1, 32) = 5.93, *p*<0.05, *η2 p* = 0.16); the low social adjustment group evoked a more negative amplitude under the congruent condition. Under the incongruent condition, there was a marginally significant difference between the high and low social adjustment groups (*F* (1, 32) = 3.57, *p* = 0.07, *η2 p* = 0.10); the low social adjustment group evoked a more negative amplitude under the incongruent condition. For the high and low social adjustment groups, there were no significant differences between the congruent and incongruent conditions (*F* (1, 32) = 3.12, *p*>0.05, *η2 p* = 0.09 and *F* (1, 32) = 0.75, *p*>0.05, *η2 p* = 0.02) (F2 in Fig 3).

**Fig 4:**
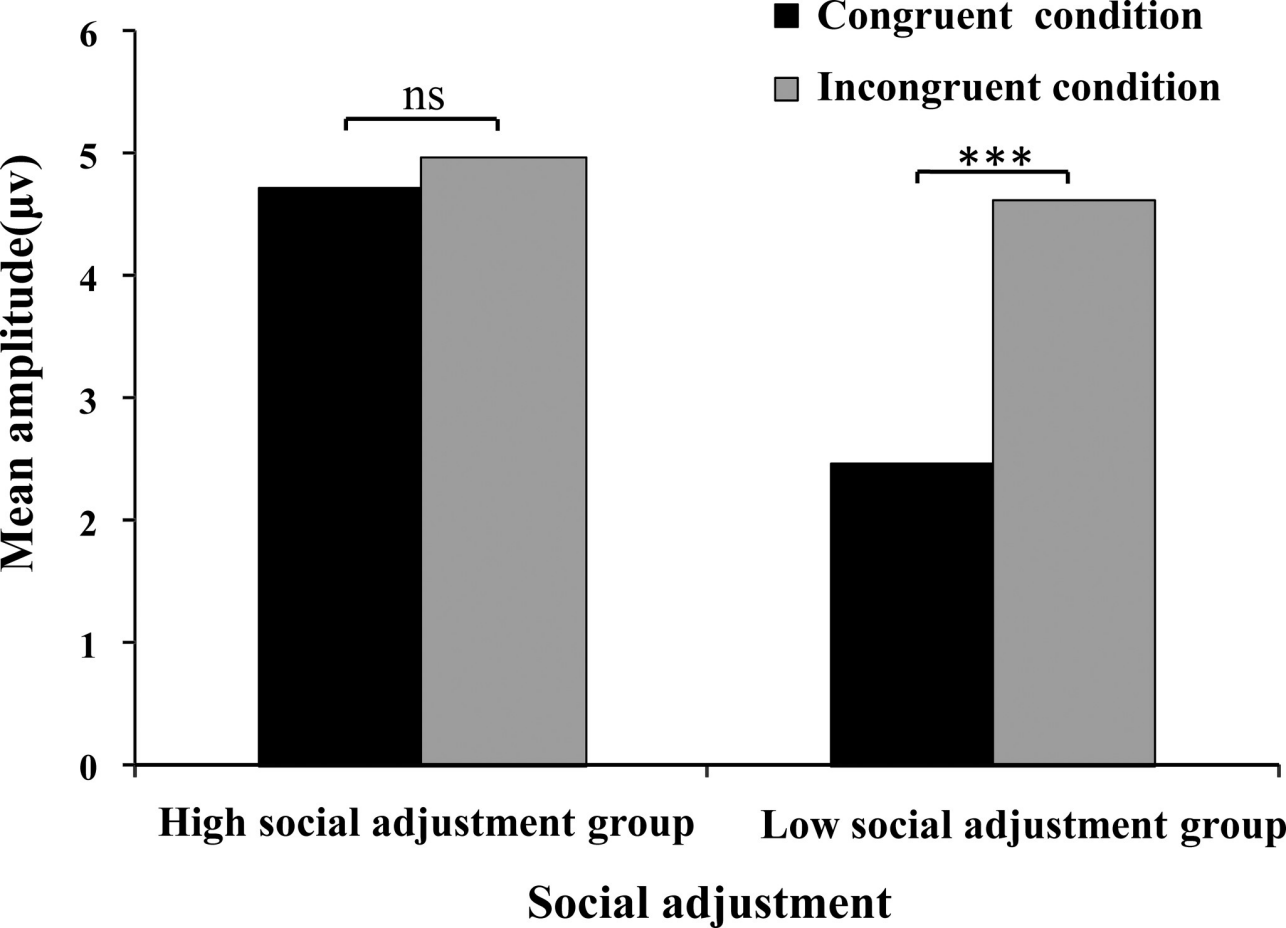
Interaction effect between social adjustment and emotional conflict for the SP component.

For the SP component, the main effect of emotional conflict was significant, *F* (1, 32) = 14.37, *p* < 0.01, *η2 p =* 0.31, while the main effect of social adjustment was not significant (*F* (1, 32) = 6.92, *p*>0.05, *η2 p = 0*.*18*). The interaction effect of emotional conflict and social adjustment was significant, *F*(1, 32) = 6.92, *p* < 0.05, *η2 p* = 0.18 (Fig 4).

Simple effects analysis indicated that under the congruent condition, there was no significant difference between the high and low social adjustment groups (*F* (1, 32) = 2.90, *p*>0.05, *η2 p* = 0.08). Under the incongruent condition, there was no significant difference between the high and low social adjustment groups (*F* (1, 32) = 0.07, *p*>0.05, *η2 p* = 0.01). For the high social adjustment group, there was no significant difference between the congruent and incongruent conditions (*F* (1, 32) = 0.64, *p*>0.05, *η2 p* = 0.02). For the low social adjustment group, there was a significant difference between the congruent and incongruent conditions (*F* (1, 32) = 21.91, *p*<0.001, *η2 p* = 0.41); the incongruent condition evoked a more positive amplitude (Poz in Fig 3).

## Discussion

ERPs were employed in this study to investigate the cognitive features and temporal characteristics of brain activity in individuals with different social adjustment levels when performing an emotional conflict task. Our behavioral results indicate that the main effect of emotional conflict was significant, such that the RT of the congruent condition was significantly faster than the incongruent condition. This is consistent with existing results [6, 20, 33], thus indicating that our experimental manipulation was valid.

The N2 component mainly reflects the process of conflict monitoring when the individual is performing the emotional conflict task [37, 38]. The N2 component is especially prominent when evoked during high-conflict tasks, such as the Stroop task and Flanker task [39]. The results of this study shown that for both the congruent and incongruent conditions, the low social adjustment group evoked more negative amplitude for the N2 component than the high social adjustment group. Under the incongruent condition of emotional conflict, there was a significant difference between the low and high social adjustment groups. This implies that compared to the high social adjustment group, individuals with low social adjustment showed poorer conflict monitoring abilities. Chen et al. [28] found that under the incongruent condition of the Stroop task, individuals are required to inhibit the interference of irrelevant stimuli, while also focusing their attention and monitor task-relevant information feature. This process involves a much higher cognitive processing load. Zhao, Shi, Fu, Cai, and Zhou [31] showed that emotion recognition and cognitive reappraisal were significantly correlated with social maladjustment, whereby social maladjustment is an important factor that causes individuals to lack the ability for emotion recognition and cognitive reappraisal. Therefore, compared to the high social adjustment group, the low social adjustment group was more susceptible to interference by the emotional Stroop task, and required more cognitive resources for conflict monitoring during the emotional conflict task, thereby evoking more negative amplitude during conflict monitoring (N2).

The SP component is a late slow wave component [27, 39] that mainly reflects the participants’ conflict processing in the emotional conflict task [29]. On the one hand, this study found that for the late slow-wave component, both the high and low social adjustment groups did not show significant differences between the congruent and incongruent conditions. A possible reason could be that during the early components, the low social adjustment group was required to devote greater attentional resources in the emotional conflict task. However, as time passed and the participants entered the late component stage, the emotional conflict was gradually resolved with the continuous increase in attentional resources [6]. This indicates that attentional resource is one of the major factors influencing emotional conflict. Hence, the attentional resources accumulated during the late component in the low social adjustment group had a facilitating effect on conflict processing. On the other hand, although the high social adjustment group did not show significant differences in the SP component between the congruent and incongruent conditions, the low social adjustment group evoked more positive amplitude in the SP component for the incongruent condition compared to the congruent condition. This indicates that the high social adjustment group showed conflict processing, whereas the low social adjustment group did not.

In summary, as an indicator of conflict monitoring, the N2 component showed that the low social adjustment group had to dedicate more cognitive resources than the high social adjustment group. That is the low social adjustment group showed greater difficulties in the conflict monitoring task. As for the SP component, which is an indicator of conflict processing, there were no differences between the congruent and incongruent conditions for both the high and low social adjustment groups. However, within-group differences indicate that the low social adjustment group did not demonstrate conflict processing, whereas the high social adjustment group did. The findings above have further enriched the theoretical research on the relationship between social adjustment and emotions [40, 41], while also providing a reference for the modification of social adjustment abilities in individuals.

## Conclusion

The difference in the emotional conflict process between individuals with high and low social adjustment mainly lies in the early processing stages of emotional information. That is, for both congruent and incongruent emotional stimuli, individuals with high social adjustment showed better emotional conflict monitoring, used less cognitive resources, and had higher degree of automated processing than those with low social adjustment. During the later stages of emotional conflict processing, individuals with low social adjustment showed poorer conflict processing.

## Research prospects and limitations

On the one hand, this study adopted the congruency of emotion between faces and words to explore the cognitive features of individuals with high and low social adjustment for emotional conflict information. Subsequent studies can adopt more complex sequential congruency effects for further exploration. On the other hand, although ERPs have a high temporal resolution, there are limitations to their spatial resolution. Therefore, fMRI should be performed in future studies to further compare the spatial characteristics of brain activity between individuals with high and low social adjustment when performing the emotional conflict task.

## Supporting information

S1 Table Mean reaction time and accuracy of face-word Stroop task (M±SD). (PDF)

S1 Dataset Data about N2 200–260. (SAV)

S2 Dataset Data about SP 700–800. (SAV)
