# Supplementary material for: Differences in the emotional conflict task between individuals with high and low social adjustment: An ERP study
Source: PLoS One. 2019 Jun 12;14(6):e0217962. doi: 10.1371/journal.pone.0217962 (PMC6561563; doi:10.1371/journal.pone.0217962)
Supplement: S1 Table — (PDF) [file pone.0217962.s001.pdf]

Table 1. Mean reaction time and accuracy of face-word Stroop task (*M*±*SD*)

Dependent variable	High social adjustment		Low social adjustment	
	Congruent	Incongruent	Congruent	Incongruent
RT (ms)	548.57 ±32.13	572.54 ±40.68	554.70 ±32.53	582.97 ±33.92
Accuracy	0.97 ±0.02	0.93 ±0.04	0.98 ±0.02	0.93 ±0.05
